# Sex Differences in Metabolic Indices and Chronic Neuroinflammation in Response to Prolonged High-Fat Diet in ApoE4 Knock-In Mice

**DOI:** 10.3390/ijms23073921

**Published:** 2022-04-01

**Authors:** Jennifer M. Mattar, Mark Majchrzak, Jaclyn Iannucci, Sydney Bartman, John K. Robinson, Paula Grammas

**Affiliations:** 1 Interdisciplinary Neuroscience Program, University of Rhode Island, Kingston, RI 02881, USA; jennifer_mattar@uri.edu (J.M.M.); jmiannucci@tamu.edu (J.I.); sydney0318@uri.edu (S.B.); 2 George & Anne Ryan Institute for Neuroscience, University of Rhode Island, 130 Flagg Rd, Kingston, RI 02881, USA; mark_majchrzak@uri.edu (M.M.); paulagrammas@gmail.com (P.G.); 3 Department of Neuroscience and Experimental Therapeutics, College of Medicine, Texas A&M Health Science Center, 8447 Riverside Pkwy, Bryan, TX 77807, USA; 4 Department of Psychology, University of Rhode Island, 130 Flagg Rd, Kingston, RI 02881, USA; 5 Department of Biomedical & Pharmaceutical Sciences, University of Rhode Island, 130 Flagg Rd, Kingston, RI 02881, USA

**Keywords:** late-onset Alzheimer’s disease, Western-pattern diet, memory, liver, neuroinflammation, diabetes, obesity, ApoE4, modifiable lifestyle factors, C57BL/6NTac mice

## Abstract

Late-onset Alzheimer’s disease (LOAD) likely results from combinations of risk factors that include both genetic predisposition and modifiable lifestyle factors. The E4 allele of apolipoprotein E (ApoE) is the most significant genetic risk factor for LOAD. A Western-pattern diet (WD) has been shown to strongly increase the risk of cardiovascular disease and diabetes, conditions which have been strongly linked to an increased risk for developing AD. Little is known about how the WD may contribute to, or enhance, the increased risk presented by possession of the ApoE4 allele. To model this interaction over the course of a lifetime, we exposed male and female homozygote ApoE4 knock-in mice and wild-type controls to nine months of a high-fat WD or standard chow diet. At eleven months of age, the mice were tested for glucose tolerance and then for general activity and spatial learning and memory. Postmortem analysis of liver function and neuroinflammation in the brain was also assessed. Our results suggest that behavior impairments resulted from the convergence of interacting metabolic alterations, made worse in a male ApoE4 mice group who also showed liver dysfunction, leading to a higher level of inflammatory cytokines in the brain. Interestingly, female ApoE4 mice on a WD revealed impairments in spatial learning and memory without the observed liver dysfunction or increase in inflammatory markers in the brain. These results suggest multiple direct and indirect pathways through which ApoE and diet-related factors interact. The striking sex difference in markers of chronic neuroinflammation in male ApoE4 mice fed the high-fat WD suggests a specific mechanism of interaction conferring significant enhanced LOAD risk for humans with the ApoE4 allele, which may differ between sexes. Additionally, our results suggest researchers exercise caution when designing and interpreting results of experiments employing a WD, being careful not to assume a WD impacts both sexes by the same mechanisms.

## 1. Introduction

A growing body of evidence supports the theory that development of late-onset Alzheimer’s Disease (LOAD) is the result of interactions between genetic predisposition and modifiable lifestyle behaviors. Multiple risk factors for Alzheimer’s disease (AD) have been identified, such as possession of the apolipoprotein E4 (ApoE4) allele, biological sex, and dietary patterns contributing to the increasing worldwide incidence of AD through complex interactions [1]. A better understanding of such interactions would help elucidate mechanisms by which LOAD develops and may inform novel therapeutic targets and prevention efforts to better treat this devastating disease.

AD shares common features of other neurodegenerative diseases (NDDs), such as neuroinflammation, neuronal loss, diminished functional and cognitive outcomes, and pathological accumulation of proteinacious substances within and surrounding neurons. In particular, the hallmark of AD is the accumulation of Amyloid-β (Aβ) and tau proteins (specifically, the pathological form of Aβ-42 and phosphorylated tau (p-tau)). The aggregation of these proteins leads to innate immune responses, such as activation of glial cells and release of pro-inflammatory cytokines, which results in subsequent neuroinflammation. This cascade of neuroinflammation leads to further neuronal damage and loss [2,3,4]. These deposits form Aβ *plaques* in the brain parenchyma surrounding neurons, while p-tau contributes to *tangles* formed within neurons, which contribute to the breakdown of cytoskeletal structure and neurodegeneration [2,5,6,7].

ApoE is a 34 kDa glycoprotein produced primarily by glial cells and by neurons (under certain conditions) in the central nervous system (CNS). ApoE plays an important role in lipid transport and response to excitotoxic injury [8,9]. Further, because ApoE binds to the Aβ peptide, it has been implicated in Aβ aggregation and clearance in the brain [8,10,11]. The human APOE gene coding for this protein, located on chromosome 19, is polymorphic and encodes three apoE protein isoforms: E2, E3, and E4. Possession of the E4 allele is currently thought to be the most significant genetic risk factor related to development of LOAD. Worldwide, 60% of AD patients possess at least one E4 allele [12]. Possession of the E4 allele differentially impacts males’ and females’ risk of developing LOAD, with 60% of AD cases being female [13,14]. In their recent cohort study, the absolute 10-year risk of AD for those with homozygous possession of the E4 allele differed for women and men and by age groups, as follows: 60–69 years, 7% and 6%; 70–79 years, 16% and 12%; and 80 years or older, 24% and 19% [15]. Although the mechanism by which ApoE4 may convey stratified risk for women is unknown, evidence suggests that it may be due to an interaction between genotype and the sex hormone estrogen [16,17,18,19].

In addition to genetics and sex, lifestyle is also implicated in LOAD [9,19,20,21]. Among lifestyle consequences, obesity is recognized as an important modifiable risk factor of LOAD in humans and has also been shown to both exacerbate and accelerate development of AD-related pathology in rodent models [22]. Obesity has been linked to metabolic syndrome, glucose hypometabolism, and insulin sensitivity or resistance [23]. In the CNS, obesity has been shown to impair the blood–brain barrier (BBB) permeability, activate microglial response, upregulate inflammatory cytokines’ expression, increase oxidative stress, and alter the structure and function of hippocampi in rodent models [24,25,26]. Diet-induced obesity has been linked to metabolic disorders, maladaptive hormonal signaling, and pro-inflammatory states, as well as reduced synaptic plasticity and neurogenesis [18,27,28].

In particular, the Western-pattern diet (WD) has been highly correlated to obesity [22,24,26]. The WD is characterized by a prolonged intake of saturated fats and sugars and is commonplace in countries adopting a Westernized lifestyle. The translation of the WD to animal studies is accomplished by dietary formulations with significantly elevated levels of saturated fats and sucrose and has revealed similar consequences to prolonged intake of this diet. The WD has been shown to yield increased effects of neuroinflammation and impair cognition in both humans and rodents [26,29,30,31,32] and has been implicated as a causal pathway to LOAD development, especially as it relates to midlife dietary patterns [33,34]. Murine models have demonstrated WD exposure results in impaired glucose tolerance [35]. Numerous studies have reported elevated biomarkers of oxidative stress and neuroinflammation in animals fed a WD [29,30,36,37]. The WD has also been associated with robust increases in amyloid accumulation and activation of glia [17,22,31,38].

Despite these well-documented findings, the interactions among genetic predisposition, sex, and a WD in the context of LOAD have been understudied. Therefore, this study aimed to explore the contributions of these risk factors in rodents in which each of these factors could be controlled. Utilizing transgenic male and female mice homozygous for the ApoE4 allele versus wild-type (WT) mice, we examined the effects of long-term exposure to a WD on metabolic variables, measures of neuroinflammation, and behavioral assays. We hypothesized exposure to a WD would result in metabolic disturbances in all animals and lead to neuroinflammation and subsequent cognitive impairment, with the greatest effects being in the ApoE4 groups.

## 2. Results

### 2.1. Genotype Does Not Affect Weight Gain

**Figure 1:**
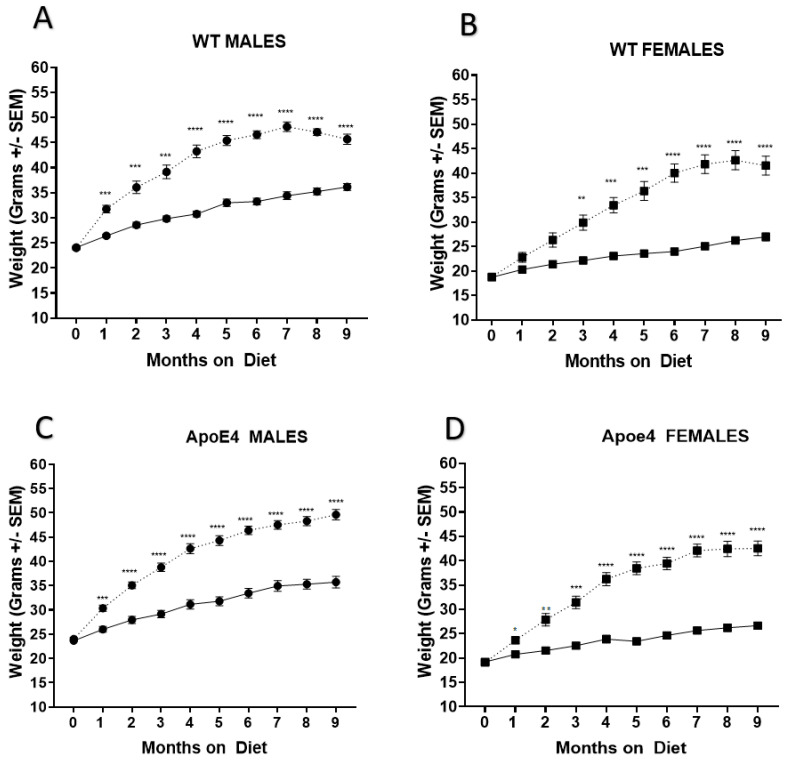
Body weights of WT and ApoE4 mice during the treatment period. Both male and female WT (**A**,**B**) and ApoE4 (**C**,**D**) mice gained weight at comparable rates throughout the study period. ^…^*WD,* -*CONTROL;* * *p* < 0.05, ** p < 0.01, *** p < 0.001, **** *p* < 0.0001.

**Figure 2:**
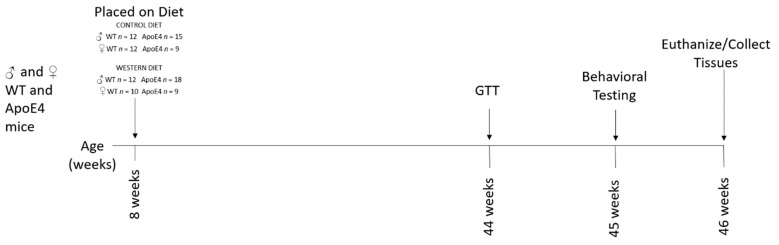
Experimental timeline (GTT = glucose tolerance testing). See Section 4: Methods for details.

Both male and female WT-fed and ApoE4 control-fed mice gained weight at comparable rates throughout the study (Figure 1). Animals on the WD gained significantly more weight than their control counterparts (WT control vs. WD: males *F*_(1, 22)_ = 107.6, *p* < 0.0001 and females *F*_(1, 21)_ = 52.02, *p* < 0.0001). ApoE4 male and female control-fed weights did not differ from WT male and female control-fed counterparts. These results suggest homozygous possession of the ApoE4 allele alone does not affect weight gain in young mice fed the WD (Figure 2).

### 2.2. WD Alters Glucose Metabolism, Especially in Female Mice

**Figure 3:**
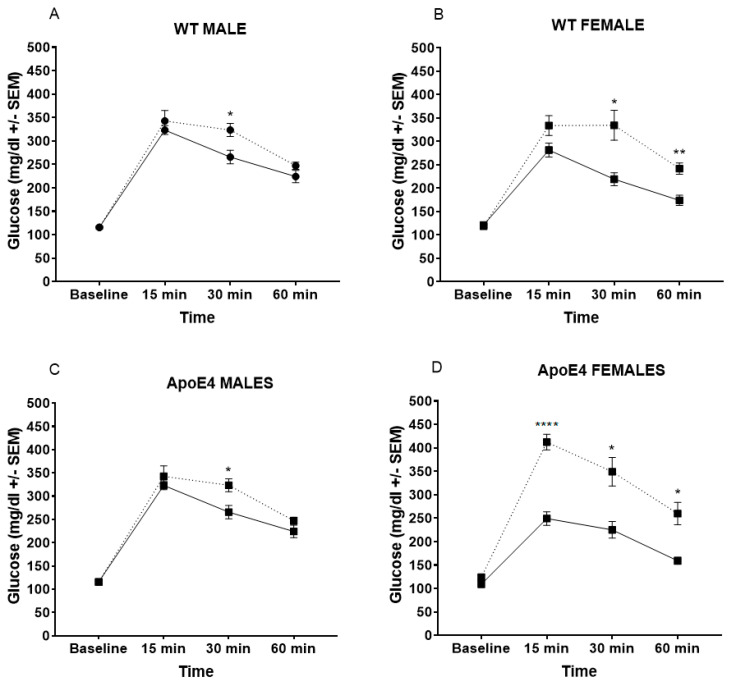
Glucose tolerance test. Average residual blood glucose levels in the 60 min period post-administration were elevated in all groups fed the high-fat diet. However, this effect was more pronounced in both female WT (**B**) and ApoE4 mice (**D**) than in either male WT (**A**) or ApoE4 (**C**) animals * *p* < 0.05, ** *p* < 0.01, **** *p* < 0.0001.

Residual glucose levels 60 min post-bolus were elevated in all WD mice (Figure 3). This effect was more pronounced in females (Figure 3B,D), regardless of genotype (WT: *F*_(1, 20)_ = 13.65, *p* = 0.0014; ApoE4: *F*_(1, 16)_= 44.64, *p* < 0.0001). Notably, we observed a marked elevated response in ApoE4 females on the WD at 15 min post-bolus (Figure 3D) that was not observed in any other group.

### 2.3. Selective Effects of WD on Liver Weights in ApoE4 Males and WT Animals

**Figure 4:**
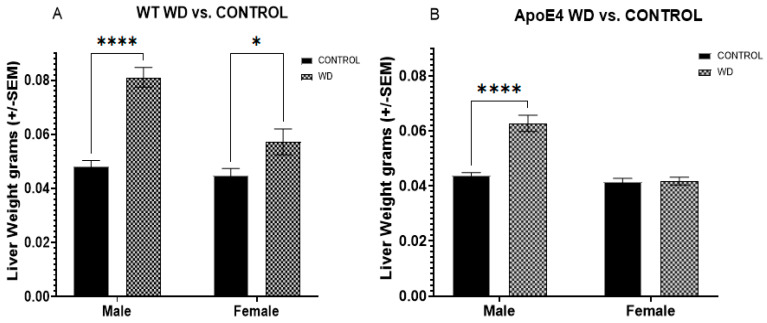
Liver weights. Male and female WT (**A**) and male ApoE4 (**B**) animals exposed to WD were found to have significantly larger livers than those on control. Female ApoE4 animals did not show this increase in liver weight. * *p* < 0.05, **** *p* < 0.0001.2.3. Selective Effects of WD on Liver Weights in ApoE4 Males and WT Animals.

Male and female WT animals exposed to the WD had significantly larger livers than those on the control diet (Figure 4A), even after correcting for body weight (*F*_(1, 42)_ = 45.93, *p* < 0.0001). Likewise, male and female ApoE4 animals exposed to the WD (Figure 4B) were found to have significantly larger livers than their control diet counterparts (*F*_(1, 47)_ = 61.40, *p* < 0.0001). However, when corrected for body weight, the effect was only significant in male ApoE4 animals (*F*_(1, 46)_ = 14.19, *p* = 0.0005) with a Bonferroni-adjusted *p* < 0.0001.

### 2.4. Reduced Glutathione Levels in WD Male ApoE4 Mice

**Figure 5:**
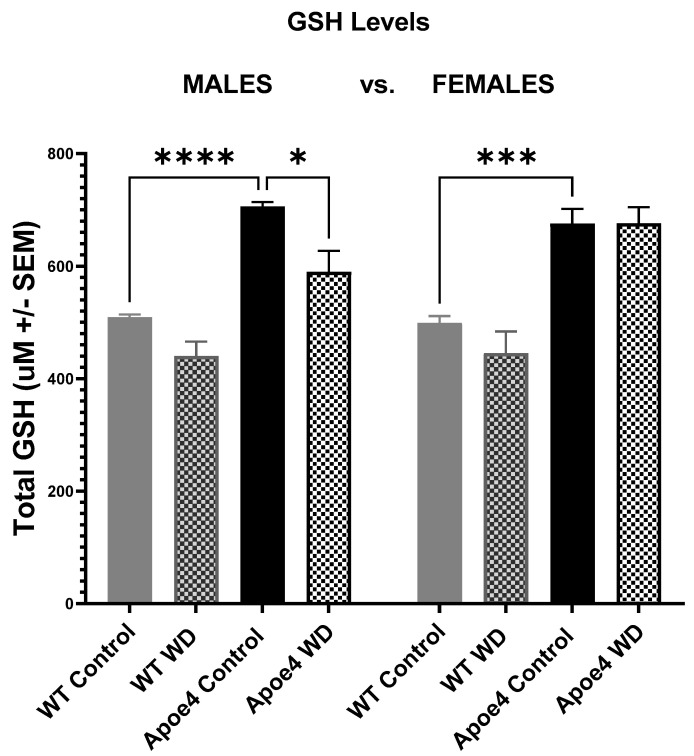
Liver glutathione levels. Baseline glutathione levels are higher in ApoE4 animals, relative to WT, and were significantly reduced only in male Western diet ApoE4 mice, (*p* < 0.05) and not females, suggesting a possible protective effect on impaired liver function and oxidative stress in these animals. * *p* < 0.05, *** *p* < 0.001, **** *p* < 0.0001.

Glutathione is a tripeptide (cysteine, glycine, and glutamic acid) which plays a crucial role in protecting cellular components from damage caused by reactive oxygen species (ROS). GSH plays a crucial role in protecting cells from oxidative damage and maintaining redox balance by directly scavenging reactive oxygen species (ROS) and other free radicals [39,40]. Reduction in GSH from the liver is an indirect measure of oxidative stress. GSH reduction in the liver has been strongly correlated with aging and neurodegeneration and occurs in the presence of progressive mitochondrial damage to mtDNA [40,41,42]. As oxidative stress in the brain increases, hepatic GSH levels drop due to an efflux of the antioxidant into the brain where it participates in redox signaling and helps restore cellular homeostatic levels [43]. Our data reveal only the interaction of ApoE4 males on the WD showed significantly decreased GSH levels when compared to their control diet counterparts (*F*_(3, 56)_ = 39.05, *p* < 0.05). No further significant sex- or genotype-based differences were found in GSH levels, although there appeared to be a trend toward lower GSH levels in both male and female WD animals when compared to those on the control diet (Figure 5). Additionally, it was noted that a significantly higher baseline mean GSH level was seen among ApoE4 animals when compared to WT animals (males *p* < 0.0001, females *p* < 0.001).

### 2.5. Interaction Effects of WD with Genotype and Sex on Measures of Neuroinflammation

**Figure 6:**
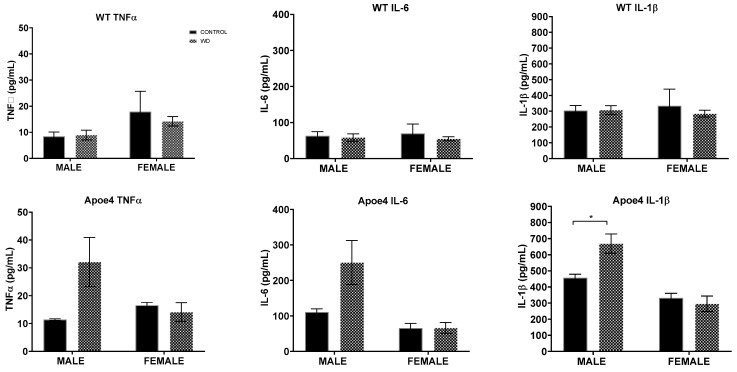
Chronic inflammatory cytokines in the brain. Expression of inflammatory cytokines was measured by ELISA. No effect of high-fat diet is evident in WT in any of the cytokines. In contrast, male (but not female) ApoE4 mice showed a significant increase in IL-1β in response to chronic exposure to the high-fat diet. * *p* < 0.05.

We investigated the expression of inflammatory cytokines TNFα, IL-6, and IL-1β in brain tissue to determine if a WD promotes neuroinflammation. We found the WD showed no significant effect on the expression of inflammatory cytokines in WT animals of either sex. However, the WD yielded a significant increase in the expression of IL-1β in ApoE4 males (*F*_(1, 12)_ = 8.002, *p =* 0.0152) compared to their control-diet-fed counterparts, but not in females (Figure 6). It should be noted that ApoE4 males on the control diet showed marginally increased expression of TNFα ( *p*= 0.0567) and IL-6 (*p* = 0.0798) when compared to control-diet-fed male counterparts.

### 2.6. Behavioral Testing Reveals Impairments from WD in Genotype Are Moderated by Sex

**Figure 7:**
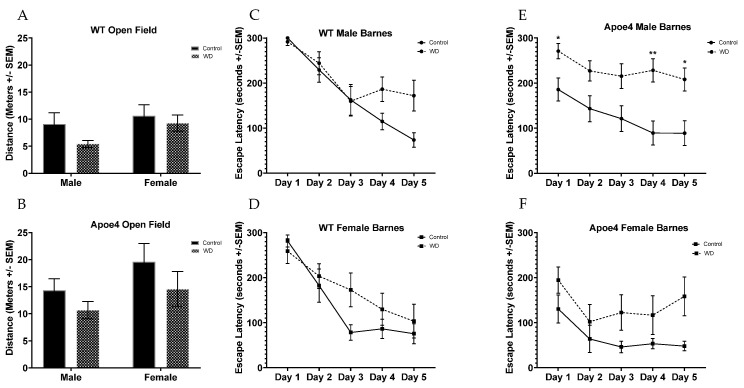
Behavioral assays. Both male and female WT (**A**) and ApoE4 (**B**) mice fed WD trended toward less activity in the open-field test. WT male and female mice fed WD (**C**,**D**) showed marginally slower latency to escape in the Barnes maze. In contrast, both male and female ApoE4 mice fed the WD (**E**,**F**) showed consistently longer latencies to escape, with this effect being significant and most pronounced in the male mice (**E**). It should be noted that control diet ApoE4 mice (**E,F**) showed consistently shorter latencies to escape than WT (**C,D**) control-diet-fed animals and non-significant differences in ambulation in the open-field where ApoE4 animals (**B**) ambulated more than WT animals (**A**), regardless of sex. * *p* < 0.05, ** p < 0.01.

In ApoE4 animals, consistently longer latency to escape was observed in WD-fed animals when compared to those on the control diet (Figure 7). This effect was significant and most pronounced in male ApoE4 WD animals (*F*_(1, 30)_ = 12.92, *p* = 0.0011). Both WT and ApoE4 animals fed a WD demonstrated decreased activity in open-field testing. However, this decreased activity could be explained by the increased body mass seen in all WD-fed animals, causing impaired ambulation or exertional effects. Though not significant (*p* = 0.11), it was observed that ApoE4 animals, particularly ApoE4 females, traveled almost twice as much in the open field as WT animals. Barnes maze testing revealed increased latency to escape that approached significance in males (*p* = 0.0558) but not in female WT animals subjected to the WD. However, both male and female ApoE4 mice fed the WD (E,F) showed consistently longer latencies to escape as compared to control-fed ApoE4 mice, with this effect reaching significance and being most pronounced in the male mice (E) (*F*(_1, 30)_ = 12.92, *p =* 0.0011). The increased ambulation in the open field also seen in this group could explain the shorter latency to escape times; as an animal’s ambulation increases, one could reason the animal will then find the escape location more quickly. A flattening of the curve for ApoE4 WD females represents a possible impairment in these animals in their ability to either learn the task or to recall the location of the fixed escape. Though, overall, the female ApoE4 animals began with markedly shorter latency to escape times than all other groups; over the course of the 5-day assay, the addition of the WD impaired the performance of the ApoE4 females and revealed an acquisition curve that actually trends towards longer latencies as day 3 through 5 of testing progress.

## 3. Discussion

There has been a recent shift in the momentum of AD research toward multifactorial risk analysis and the subsequent combined effects on pathology progression. Separately, the effects of each individual risk factor are indeed important; however, the interaction of several risk factors (ApoE4 carrier status, sex differences, dietary patterns) has become of particular interest to understanding causal pathways of LOAD [44,45]. There is a dearth of knowledge on the effects of genetic risk compounded with modifiable lifestyle factors. Our study aimed to understand the interaction effects of these factors in a mouse model of LOAD.

We found that alterations in glucose metabolism, liver function, and inflammatory cytokine expression were observed as complex interactions. These alterations related to behavioral impairments, with sex and diet moderating the relationship between genotype and both metabolic and cognitive outcomes. These findings are consistent with an extensive body of research on the effects of a high-fat diet in mice [37,38,46,47], where male mice show consistent detrimental effects on neuroinflammation, glucose tolerance, and behavioral measures [48,49,50]. In humans, males are at increased risk of developing metabolic syndrome (MetS), which is associated with obesity [51,52]. ApoE4 possession has also been linked to increased risk of MetS in a dose-dependent manner as BMI increases [25]. Similarly, rodent models have demonstrated that possession of ApoE4 increases risk of metabolic syndrome, possibly by interfering in insulin-sensing pathways [25,53]. In European women under 80, the prevalence of impaired glucose tolerance is higher [54]. We have shown that a WD alters glucose metabolism in both WT and LOAD animals, especially in females, with the most profound effect occurring in ApoE4 females. Remarkably, despite experiencing the most exaggerated response to GTT, ApoE4 females fed the WD showed no effects on liver weight or GSH levels. Both male and female WT animals fed the WD exhibited significantly larger livers than their control-fed counterparts. In ApoE4 animals, males fed WD also yielded significantly larger livers with female ApoE4 animals appearing to be spared from this effect. Additionally, despite the significant increase in liver weight seen in ApoE4 males fed WD, the ApoE4 males’ liver weight response to the WD was less pronounced than that of the WT males. While WD exposure resulted in minimally decreased levels of GSH in the livers of WT animals, the only significant reduction observed was again in ApoE4 males. This inverse relationship of elevated liver weight and decreased GSH levels in WD ApoE4 males is particularly interesting, since this group also yielded findings of elevated markers of neuroinflammation and the poorest performance in the Barnes maze. Oxidative stress and neuroinflammation have been linked to early biochemical changes associated with the progression of AD [55] and could explain the severe impairment seen with Barnes maze.

What is not clear is the explanation for female ApoE4 WD animals demonstrating a null effect in liver weight, GSH reduction in the liver, and inflammatory cytokine expression in the brain, yet revealing impairment in cognition as illustrated by the flattened curve in Barnes performance in this group. Numerous studies in both humans and rodents have shown sex-dependent effects on LOAD risk and pathology severity [12,35,45]. However, the literature on humans offers mixed explanations for these observations, with many supporting the simple notion that longer female life expectancy confers increased age-related risk, while some epidemiologic reports cite “survival bias” resulting from the healthiest aged men surviving to older ages and driving down AD occurrence in males [56]. Some studies have suggested the ApoE4 genotype may have a stronger relationship with development of AD in women versus men [45,56,57], perhaps due to hormone effects.

Age and sex interact with ApoE4 and obesity to combine for increased detrimental effects on metabolism and cognition [58]. Although the underlying explanation for these observations remains to be elucidated, several explanations have been offered, such as differential impacts of aging, sex differences in lean muscle mass, adipocyte distribution, influence of the estrous cycle and menopause, and invoked susceptibility to free-fatty acid-induced peripheral insulin resistance [54]. Human males and females show distinct dimorphism and substantial variability in adipocyte distribution that may be exclusive to our species [53]. Adipocyte size is important in determining its function and metabolic activity, regardless of obesity level, and is closely correlated to insulin resistance, dyslipidemia, and triglyceride synthesis [54]. The location of adipose tissue can be either subcutaneous (SAT) or visceral (VAT), and each type relates differently to development of Mets across an aging continuum. VAT is closely correlated to metabolic disturbances and cardiovascular risk [59,60]. Males tend to develop more VAT, which remains stable across a lifetime but responds better to overall weight reduction, whereas females tend to develop more SAT, which has been reported to show protective effects [61,62]. In one study, premenopausal women were observed to accumulate a substantial amount of total body fat before increases in VAT were noted [63]. Postmenopausal women accumulate more VAT, which increases in size due to estrogen-depletion-induced hyperplasia [63]. In one recent study, female ApoE4 mice subjected to ovarian failure revealed cognitive impairment and extensive deficits in synaptic-plasticity-related signaling [58].

Taken together, these findings may suggest a mechanism where males with possession of both ApoE4 alleles may be more susceptible to the deleterious metabolic effects of prolonged exposure to a WD. We suggest that in males homozygous for ApoE4, a WD leads to metabolic dysfunction that results in an increase in liver weight, impaired liver function, and impairment in glucose metabolism. Liver dysfunction and altered glucose metabolism yield an increase in pro-inflammatory cytokine expression in the brain, thereby contributing to oxidative stress and neurodegeneration and leading to cognitive impairment. In female ApoE4 mice chronically exposed to a WD, no such metabolic changes were observed except for the exaggerated response to glucose tolerance. Although ApoE4 females did arrive at the similar endpoint of impaired learning and memory as demonstrated in behavioral testing on the Barnes maze, there is no clear causal pathway that emerged from our findings. Therefore, we must be careful not to assume these effects were simply due to an altered metabolic response as indicated by the GTT results, as there were no changes in inflammatory cytokine expression in the brains of these animals. Further investigation is needed to expose a possible alternative explanation as to the mechanism by which ApoE4 females fed a WD become cognitively impaired over time.

It is possible, even likely, that adipocyte distribution and size differences between the sexes, combined with hormonal signaling effects of substances such as estrogen and leptin (which are stored in these adipocytes), may play a crucial role in understanding how the moderating effects of sex and diet on ApoE4 carry status and risk of LOAD. Future research should focus on investigating potential sex-specific factors, such as estrogen, that may moderate the relationship between the WD and development of LOAD in females carrying both E4 alleles. One important point to consider when seeking to elucidate these mechanisms is the age of animals, particularly as they relate to the estrous cycle. Extensive literature reviews revealed a body of research focusing primarily on either younger or older models. Our study was conducted using a one-year time point (after 9 months on diet), which equates to a mid-life paradigm in C57BL/6J mice. After 6 months of age, mice mature at a rate 25 times that of humans. Therefore, our 12-month timepoint correlates to humans 38–47 years old [64,65]. It is likely that possession of ApoE4 affects females differentially across an aging continuum, and future studies should seek to probe into WD effects in aged mice at later timepoints.

It is also interesting to consider what our findings say about using a WD manipulation to invoke metabolic syndrome in mice. Despite resulting in obesity in all groups of treated mice, we observed a clear dissociation between the liver vs. glucose regulation consequences in our mice. This suggests that investigators should be cautious in assuming a WD produces a unitary metabolic syndrome-like pattern of effects in mouse models. Importantly, studies have shown male mice are more vulnerable than female mice to deleterious effects of a WD on weight, metabolic alterations, and deficits of learning, and hippocampal synaptic plasticity [58,66]. Further research will need to elucidate the specific potential divergent casual pathways, particularly the sex differences, on human ApoE4 risk in relation to diet. Our research raises the probability that precision medicine approaches may be of great benefit to targeting the prevention and treatment of AD, especially as sex differences and timing of intervention may differ greatly for individuals homozygous for ApoE4.

## 4. Limitations

A limitation of our study was the use of whole brain homogenate to assess for markers of neuroinflammation. Our study did not utilize sectioning or immunohistochemistry/staining techniques to look for accumulation of AD biomarkers (Aβ, p-tau), nor did we look at markers of glial/immune activation, neurodegeneration, necrosis, or autophagy. Additionally, we did not examine the VAT pads postmortem in our mice, which would have been helpful to better understand the relationship of overall weight gain versus the increase in VAT between the sexes. Future studies should consider measuring these biomarkers and tissues and examine specific regions, such as the hippocampi and medial temporal lobes, frontal lobe, entorhinal cortex, and ventromedial prefrontal cortex (vmPFC), areas shown to be involved in learning and memory, accumulate protein aggregates, and show neurodegeneration, and are thought to be functionally connected [67,68,69]. Since our study looked at “middle-aged” mice, future studies should seek to replicate our findings and add additional aging timepoints (perhaps at additional 3-month intervals) to follow the progression of continued exposure to a WD to potentially observe a difference in female ApoE4 mice at later points as estrogen levels decline.

## 5. Materials and Methods

### 5.1. Animals

Transgenic mice expressing human ApoE4 ^+^/^+^ (C57BL/6NTac-Apoe < tm4207.1, APOE*R130, *R176) and wild-type (WT) controls (C57BL/6NTac) were purchased from Taconic Biosciences, Inc. (Rensselaer, NY). Mice were aged eight weeks, then were maintained on either normal chow (ENVIGO TD2014, 14% protein, Indianapolis, IN) or WD (ENVIGO TD81337, adjusted-calorie diet with 42% kcal from fat) with water available ad libitum. All animals were housed in a temperature-controlled room (22 ± 2 °C and 40–60% humidity) on a standard 12 h light cycle. At 46 weeks of age, following metabolic and behavioral testing, mice were deeply anesthetized with I.P. administration of ketamine (80 mg/kg) and xylazine (10 mg/kg) and euthanized via terminal cardiac puncture. Animal blood, brain, and liver tissue was collected for further analysis. All animal procedures were performed in accordance with NIH’s *Guide for the Care and Use of Laboratory Animals* and University of Rhode Island’ Animal Care and Use Committee’s (IACUC) guidelines.

### 5.2. Glucose Tolerance Testing (GTT)

At 44 weeks of age, one week prior to behavioral testing, animals underwent oral glucose tolerance testing (GTT). After a six-hour fast, baseline blood glucose was measured vial lateral tail vein puncture using an AlphaTrak 2 glucose testing system (Zoetis, Parsippany, NJ, USA). Animals were dosed via oral gavage, with a 2 g/kg dose of anhydrous D(+) glucose (Sigma Aldrich, St. Louis, MO, USA) mixed with sterile water and given a 1 mL/kg bolus, according to weight. Blood glucose levels were measured post-gavage at 15, 30, and 60 min, respectively.

### 5.3. Behavioral Assays

#### 5.3.1. Open Field

The Open-Field test was conducted to assess general locomotor activity, exploratory behavior, and anxiety-like behavior at 45 weeks of age. The apparatus (Stoelting Co., Wood Dale, IL, USA) comprised a gray, non-reflective, powder-coated metal base (40 cm^2^) surrounded by gray, opaque walls (35 cm high). Animals were placed in the center of the open field and allowed to explore the area for 10 min, during which time the number of animal rears were manually scored by the experimenter while general locomotion activity (i.e., speed and distance travelled) was automatically recorded by AnyMaze^TM^ behavioral tracking software (version 6.1, Stoelting Co., Wood Dale, IL, USA). Rearing was defined as an animal lifting off from its front paws and placing all its weight on its hind paws, including when assisted by a wall.

#### 5.3.2. Barnes Maze

The Barnes maze was conducted to assess spatial learning and memory. The maze (Stoelting, Co., Wood Dale, IL, USA) was made from a circular, gray, non-reflective, powder-coated metal base platform with a diameter of 91 cm. Twenty holes, each with a diameter of 5 cm, surrounded the perimeter of the maze at 2.5 cm from the edge. One hole was designated as the escape location and a 7.5 × 15 × 5 cm goal box was situated beneath the hole to allow the animal a point of egress and seclusion. Testing occurred each morning at the same time of day over five days. Prior to each trial, animals were acclimated to the testing room for 15 min. During testing, animals were placed inside a 10 × 10 × 20 cm plastic, semi-opaque acclimation container in the center of the maze for 20 s. Then, the container was removed, and the animal was allowed to explore the maze for a maximum of 300 s, or until they successfully found the escape hole. Animals remained (or were placed inside) the escape box with the hole covered for one minute to reinforce the location of escape. After one minute, animals were returned to their home cage. Following completion of each trial, the maze and escape box were thoroughly cleaned with a solution of 70% ethanol to mask olfactory cues from previous trials.

The Barnes maze and open-field apparatus were placed on top of a platform, 80 cm above the ground, and made level. The platform was situated in the middle of a dedicated room, as far away from the investigator as possible, under a fixed ceiling lighting apparatus. Extra-maze visual cues were fixed at eye-level for the animals and remained constant for every cohort. All activity was recorded using a Logitech C270 web camera mounted 1.5 m above the maze, and AnyMaze^TM^ behavioral tracking software was used for data collection.

### 5.4. Enzyme-Linked Immunosorbent Assay (ELISA)

Levels of inflammatory cytokines tumor necrosis factor α (TNFα), interleukin (IL)-6, and IL-1β were measured in whole-brain homogenate using Enzyme-Linked Immunosorbent Assay (ELISA) from Biolegend (San Diego, CA, USA); mouse IL-1β cat.

#432604, Mouse TNFα cat. #430904, Mouse IL-6 cat. #431304) in 3–5 mice drawn randomly from each test group. The protocol provided by the manufacturer was followed, without modifications. At the conclusion of the assay, absorbance was detected at 570 nm and 450 nm using Synergy HTX multi-mode reader (Biotek Instruments, Winooski, VT, USA).

### 5.5. GSH ELISA

The levels of reduced glutathione (GSH) were assessed in liver homogenate using a Glutathione Colorimetric Detection Kit (Invitrogen), according to manufacturer’s instructions. Briefly, whole liver was sectioned and homogenized by sonication (Branson SX150 Sonifier^®^, Branson Ultrasonics, Danbury, CT, USA) in phosphate-buffered saline (PBS) with 1× protease inhibitors. Samples were further diluted according to kit instructions, added to a 96-well microplate, then incubated with reaction mixture for 20 min at room temperature. Following incubation, samples were read for absorbance at 405 nm using Synergy HTX multi-mode reader (Biotex Instruments, Winooski, VT, USA).

### 5.6. Statistical Analysis

All tests were performed in GraphPad Prism, version 9.1.0 (GraphPad, CA). Data are represented as mean +/− standard error (SEM). ELISA analysis was conducted using analysis of variance (ANOVA) with Bonferroni’s correction for multiple comparisons with group *N =* 3–5. Behavioral measures assessed for differences between groups using two-way and mixed-model ANOVA with either Tukey’s (open field) or Bonferroni’s (weights, GTT, Barnes maze, liver weights) multiple comparisons tests for post hoc discovery. Statistical significance was set at *p* < 0.05, and group *N* is listed in Figure 2 for the behavioral testing.

## Data Availability

The data presented in this study are available on request from the corresponding author.

## Abbreviations

Aβ	amyloid β-protein
ANOVA	analysis of variance
AD	Alzheimer’s disease
CNS	central nervous system
ELISA	enzyme-Linked immunosorbent assay
GSH	glutathione
GTT	glucose tolerance test
LOAD	Late-onset Alzheimer’s disease
MetS	metabolic syndrome
NDDs	neurodegenerative diseases
OF	open field
SAT	subcutaneous adipose tissue
VAT	visceral adipose tissue
WD	Western-pattern Diet
WT	wild-type
